# Residual Atherogenic Dyslipidemia in Normal-Weight Adults: The Role of Visceral Adiposity and Metabolic Dysfunction

**DOI:** 10.3390/medsci14060655

**Published:** 2026-10-09

**Authors:** Diego González Carrasco, Pedro Juan Tárraga López, Mónica Silu Piña Dabreu, Lluis Rodas Cañellas, Ángel Arturo López-González, José Ignacio Ramírez-Manent

**Affiliations:** 1 ADEMA-University School, University of the Balearic Islands, 07009 Palma, Spainm.pinadabreu@eua.edu.es (M.S.P.D.);; 2 Faculty of Medicine, University of Castilla La Mancha (UCLM), 02008 Albacete, Spain; pjtarraga@sescam.jccm.es; 3 Balearic Islands Health Service, 07010 Palma, Spain

**Keywords:** obesity, dyslipidemias, visceral fat, cardiovascular diseases, insulin resistance, body mass index

## Abstract

**Background.** Body mass index (BMI) remains the most widely used tool for obesity classification and cardiometabolic risk stratification. However, increasing evidence suggests that normal BMI does not necessarily reflect metabolic health, particularly in individuals with hidden visceral adiposity and residual atherogenic dyslipidemia. The present study aimed to evaluate the prevalence of residual atherogenic dyslipidemia among normal-weight adults and to analyze its association with visceral adiposity, metabolic dysfunction, and lifestyle-related factors. **Methods.** A cross-sectional observational study was conducted in 166,008 Spanish workers aged 18–69 years with normal BMI (18.5–24.9 kg/m^2^) who underwent standardized occupational health examinations between 2022 and 2024. Anthropometric, biochemical, vascular, and lifestyle-related variables were evaluated. Visceral adiposity was estimated using waist-to-height ratio (WtHR) and the Metabolic Score for Visceral Fat (METS-VF). Residual atherogenic dyslipidemia was primarily assessed using the triglyceride-to-HDL cholesterol ratio (TG/HDL-c). Multivariable logistic regression analyses were performed to identify independent associations with elevated TG/HDL-c. **Results.** Despite normal BMI values, 8.0% of participants presented with an elevated TG/HDL-c ratio, 11.8% had central adiposity, 11.4% showed low HDL cholesterol, and 17.9% exhibited elevated blood pressure. Individuals with an elevated TG/HDL-c ratio demonstrated significantly higher waist circumference, WtHR, METS-VF, triglycerides, non-HDL cholesterol, fasting glucose, blood pressure, and TyG index values (all *p* < 0.001). A progressive worsening of lipid and metabolic parameters was observed across increasing METS-VF quartiles. Elevated TG/HDL-c ratio prevalence increased from 2.7% in the lowest METS-VF quartile to 15.8% in the highest quartile. Men showed a substantially higher prevalence of residual atherogenic dyslipidemia than women (12.2% vs. 3.9%, *p* < 0.001). Physical inactivity, smoking, poor Mediterranean diet adherence, and regular alcohol consumption were strongly associated with adverse lipid-metabolic profiles. In multivariable analyses using separate adiposity measures, BMI and METS-VF remained independently associated with elevated TG/HDL-c, whereas WtHR ≥ 0.50 was not independently associated after adjustment. Male sex, physical inactivity, smoking, impaired fasting glucose, elevated blood pressure, and low Mediterranean diet adherence showed consistent associations across models. **Conclusions.** Residual atherogenic dyslipidemia is highly prevalent among adults with normal BMI and is strongly associated with visceral adiposity, metabolic dysfunction, and unfavorable lifestyle-related behaviors. Conventional BMI classification alone substantially underestimates hidden cardiometabolic risk. These findings highlight the metabolic heterogeneity present among apparently healthy normal-weight individuals and the limitations of BMI as a sole marker of cardiometabolic health.

## 1. Introduction

Body mass index (BMI) has traditionally been used as the principal anthropometric criterion for defining obesity and estimating cardiometabolic risk in both clinical practice and epidemiological research. Owing to its simplicity, low cost, and reproducibility, BMI remains deeply integrated into current preventive strategies and international clinical guidelines [1]. Nevertheless, growing evidence accumulated over the last two decades has demonstrated that BMI alone provides only a limited representation of adiposity-related biological risk, as it does not adequately reflect body fat distribution, adipose tissue functionality, ectopic fat deposition, or metabolic heterogeneity among individuals with similar body weight [2,3].

This limitation has become increasingly relevant with the recognition of metabolically adverse phenotypes among adults classified as normal weight according to conventional BMI thresholds [4,5]. A substantial proportion of apparently lean individuals may already exhibit insulin resistance, atherogenic dyslipidemia, endothelial dysfunction, low-grade inflammation, and increased cardiovascular vulnerability despite remaining within the normal BMI range [6,7]. Conversely, some individuals with increased adiposity may temporarily preserve relatively favorable metabolic profiles, further emphasizing the heterogeneous nature of obesity-related disease [8].

In recent years, the concept of adiposity–metabolic discordance has emerged as a useful framework for understanding the biological dissociation between body size and cardiometabolic dysfunction [9]. This paradigm recognizes that adiposity accumulation and metabolic deterioration do not necessarily evolve in parallel and that clinically relevant cardiometabolic abnormalities may occur in the absence of overt obesity [10]. Such discordance appears to be particularly relevant within the normal-weight population, where traditional anthropometric screening may underestimate hidden cardiovascular and metabolic risk [11].

Among the different manifestations of cardiometabolic dysfunction, atherogenic dyslipidemia occupies a central role because of its close relationship with insulin resistance, visceral adiposity, endothelial injury, and accelerated atherosclerosis [12]. Atherogenic dyslipidemia is typically characterized by elevated triglycerides, reduced high-density lipoprotein cholesterol (HDL-c), predominance of small dense low-density lipoprotein particles, and increased remnant lipoproteins, all of which contribute to residual cardiovascular risk independently of LDL cholesterol concentrations [13,14]. Importantly, these abnormalities may develop even in subjects without overt obesity or markedly elevated total cholesterol levels [15].

Recent evidence has increasingly highlighted the concept of residual atherogenic risk, referring to the persistence of cardiovascular risk despite apparently acceptable conventional lipid parameters [16]. This residual risk is strongly influenced by triglyceride-rich lipoproteins, lipoprotein particle composition, insulin resistance, chronic inflammation, and visceral adiposity [17]. Consequently, individuals classified as normal weight according to BMI may still harbor clinically relevant lipid abnormalities associated with early atherogenesis and long-term cardiovascular disease [18].

Visceral adipose tissue appears to play a particularly important role in this process. Unlike subcutaneous fat depots, visceral adiposity exhibits greater lipolytic and inflammatory activity, promoting increased free fatty acid flux to the liver, hepatic insulin resistance, altered lipoprotein metabolism, oxidative stress, and chronic low-grade inflammation [19,20]. These mechanisms contribute directly to the development of hypertriglyceridemia, reduced HDL-c concentrations, increased remnant particles, and small dense LDL predominance, thereby linking visceral fat accumulation with atherogenic dyslipidemia and cardiovascular disease progression [21].

Importantly, several studies have demonstrated that visceral adiposity may remain clinically underestimated in individuals with normal BMI values [22]. In this context, waist-related anthropometric markers and novel adiposity indices have shown greater ability than BMI to identify hidden cardiometabolic dysfunction [23]. Waist-to-height ratio (WtHR), in particular, has consistently demonstrated superior predictive performance for cardiometabolic abnormalities and cardiovascular risk compared with BMI alone [24,25]. Likewise, the Metabolic Score for Visceral Fat (METS-VF) has emerged as a promising surrogate marker integrating anthropometric and metabolic information to estimate visceral adiposity and adipose tissue dysfunction using routinely available clinical variables [26,27].

Parallel to the growing interest in visceral adiposity, increasing attention has been directed toward lipid-derived cardiometabolic indices capable of identifying early atherogenic alterations beyond conventional lipid measurements [28]. Ratios such as triglyceride-to-HDL cholesterol (TG/HDL-c), total cholesterol-to-HDL cholesterol, and non-HDL cholesterol have demonstrated close associations with insulin resistance, metabolic syndrome, vascular dysfunction, and cardiovascular events [29,30]. These markers may therefore facilitate the identification of residual cardiometabolic risk among apparently healthy normal-weight adults.

Lifestyle-related factors further modulate this complex interaction between adiposity, lipid metabolism, and cardiometabolic dysfunction. Physical inactivity, smoking, and poor dietary quality have all been independently associated with visceral adiposity accumulation, insulin resistance, endothelial dysfunction, and atherogenic lipid profiles even in the absence of overt obesity [31,32,33]. Mediterranean dietary patterns and regular physical activity, on the other hand, appear to exert protective effects on lipid metabolism, inflammatory pathways, and vascular health independently of BMI [34,35].

From a public health perspective, the identification of hidden atherogenic risk among normal-weight individuals has become increasingly important because reliance on BMI-based classification alone may delay preventive interventions until overt cardiometabolic disease becomes clinically established [36]. This limitation has recently prompted calls for more comprehensive approaches to obesity and cardiometabolic risk characterization incorporating adiposity distribution, metabolic dysfunction, and lipid-related residual risk [37,38].

Despite increasing interest in obesity heterogeneity and residual cardiovascular risk, evidence specifically evaluating residual atherogenic dyslipidemia among adults with normal BMI remains limited, particularly in large occupational cohorts. Moreover, the relationship between visceral adiposity, metabolic dysfunction, and lipid-related cardiometabolic risk within normal-weight populations has not yet been fully clarified.

Therefore, the present study aimed to evaluate residual atherogenic dyslipidemia among adults with normal BMI in a large occupational cohort and to analyze its association with visceral adiposity, metabolic dysfunction, and lifestyle-related factors.

## 2. Materials and Methods

### 2.1. Study Design and Population

A cross-sectional observational study was conducted using data obtained from periodic occupational health assessments performed in several Spanish autonomous communities between January 2022 and December 2024. The investigation was developed within the framework of routine workplace preventive medicine programs carried out by accredited occupational health services collaborating with ADEMA University School.

The initial database included 418,343 actively employed adults aged between 18 and 69 years who underwent standardized medical examinations during the study period. For the present analysis, only individuals classified as normal weight according to World Health Organization BMI criteria (18.5–24.9 kg/m^2^) were considered eligible [39]. Subjects with incomplete anthropometric or biochemical information, pregnancy, known cardiovascular disease, active oncological disease, severe chronic inflammatory disorders, or lipid-lowering treatment were excluded to minimize potential confounding related to advanced cardiometabolic disease or pharmacological interference.

**Figure 1:**
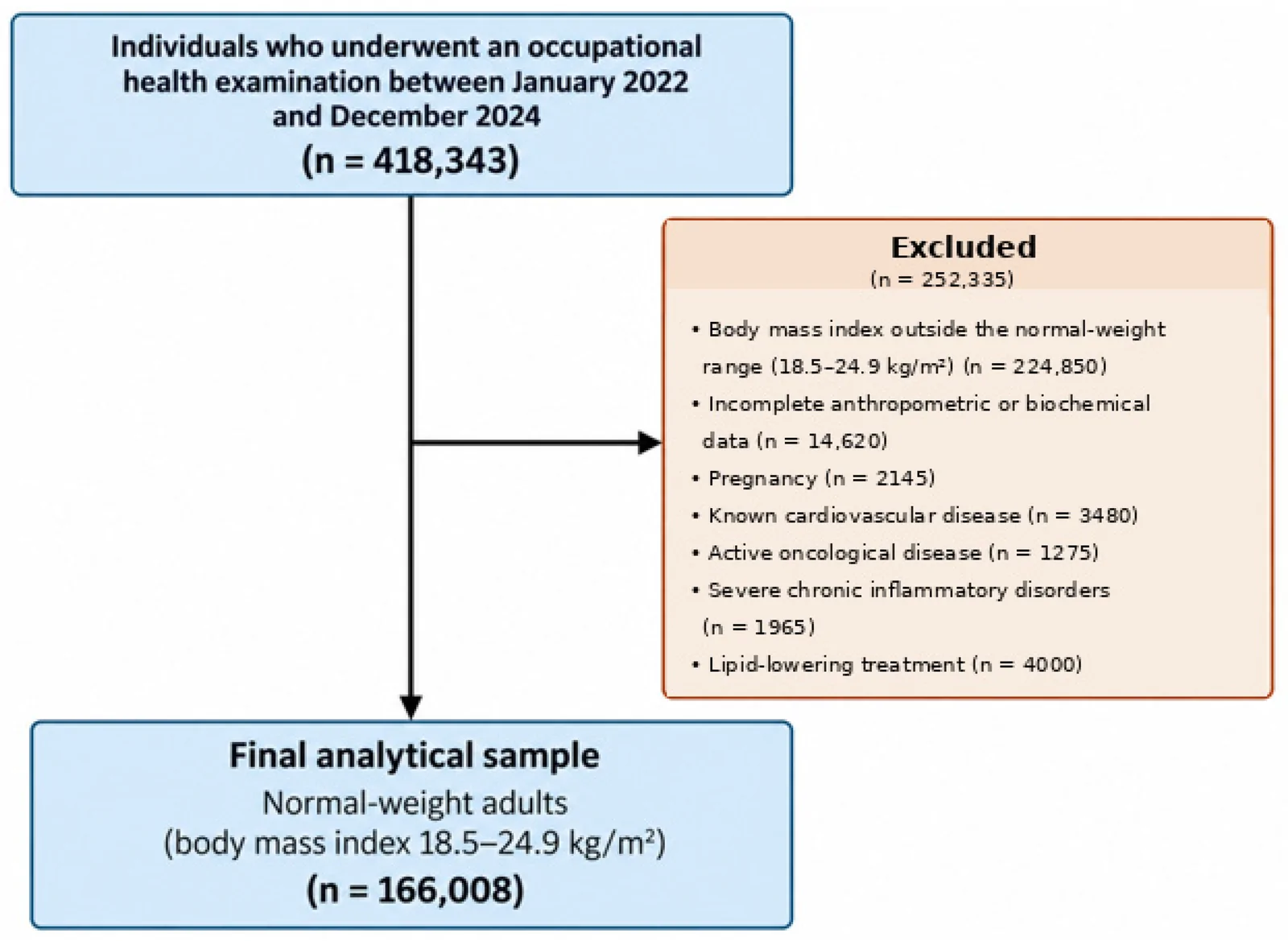
Flowchart of participant selection and study inclusion process.

After applying all exclusion criteria, the final analytical sample consisted of 166,008 workers of both sexes. The participant selection process is summarized in Figure 1.

The study followed the recommendations proposed for observational epidemiological studies and occupational population research [40,41].

### 2.2. Ethical Considerations

All procedures were performed according to the ethical standards established in the Declaration of Helsinki and its subsequent revisions [42]. The study protocol was reviewed and approved by the institutional ethics committee affiliated with ADEMA University School.

Prior to participation, all workers received detailed information regarding the characteristics and objectives of the occupational medical evaluation and provided written informed consent authorizing the anonymous scientific use of their data. To ensure confidentiality, all personal identifiers were removed before statistical processing, and data handling complied with the requirements established under European Union Regulation 2016/679 and Spanish Organic Law 3/2018 concerning personal data protection. The study protocol was evaluated and approved by the Ethics Committee of the Balearic Islands (CEI-IB; approval code IB 4383/20) on 26 November 2020.

### 2.3. Anthropometric Assessment

Anthropometric measurements were obtained by trained occupational health professionals following standardized international procedures designed to reduce interobserver variability [43]. Body weight was measured with participants barefoot and wearing light clothing using calibrated electronic scales with 0.1 kg precision. Height was determined using portable stadiometers with subjects standing upright in the Frankfurt anatomical plane.

Body mass index was calculated as weight divided by squared height (kg/m^2^). Waist circumference was measured midway between the lower rib margin and the iliac crest at the end of gentle expiration using non-extensible anthropometric tape.

Waist-to-height ratio (WtHR) was calculated by dividing waist circumference by height, both expressed in centimeters. Based on previous cardiometabolic risk studies, a WtHR value ≥ 0.50 was considered indicative of increased central adiposity [43,44].

Visceral adiposity estimation was additionally assessed using the Metabolic Score for Visceral Fat (METS-VF), a validated surrogate index integrating anthropometric and metabolic parameters associated with visceral fat accumulation [45]. METS-VF was calculated using the original equation proposed by Bello-Chavolla et al. [45].

Blood pressure was measured after at least five minutes of seated rest using validated automatic sphygmomanometers. Two determinations were obtained in each participant, and the mean value was used for analysis [46].

### 2.4. Biochemical Determinations

Venous blood samples were collected in the morning after a minimum fasting period of 12 h. All analyses were performed in certified clinical laboratories participating in external quality assurance programs.

Serum concentrations of total cholesterol, triglycerides, HDL cholesterol, LDL cholesterol, and fasting plasma glucose were determined using standardized enzymatic methods following routine clinical laboratory protocols [47].

To characterize residual atherogenic dyslipidemia, several lipid-derived cardiometabolic indices were calculated:

- triglyceride-to-HDL cholesterol ratio (TG/HDL-c),
- total cholesterol-to-HDL cholesterol ratio (TC/HDL-c),
- LDL cholesterol-to-HDL cholesterol ratio (LDL-c/HDL-c),
- and non-HDL cholesterol.

Non-HDL cholesterol was obtained by subtracting HDL cholesterol from total cholesterol values. These indices have demonstrated close relationships with insulin resistance, endothelial dysfunction, visceral adiposity, and cardiovascular risk in both clinical and epidemiological studies [48,49,50].

For the primary operational analysis, residual atherogenic dyslipidemia was defined as a TG/HDL-c ratio ≥ 3.0. The TG/HDL-c ratio was selected as the primary operational marker to capture a broader atherogenic-metabolic phenotype characterized by the combined balance between triglyceride and HDL-c concentrations. This operational definition was analyzed separately from classic atherogenic dyslipidemia.

Classic atherogenic dyslipidemia was defined separately according to previously established criteria requiring the concomitant presence of elevated triglyceride concentrations and reduced HDL cholesterol levels [51].

### 2.5. Lifestyle-Related Variables

Lifestyle information was collected through structured health questionnaires routinely administered during occupational medical examinations.

Smoking status was originally recorded as current smoker, former smoker, or never smoker. For the present analyses, smoking status was dichotomized as current smokers versus non-smokers, with former and never smokers included in the non-smoker category.

Physical activity was assessed using the International Physical Activity Questionnaire (IPAQ). For the present analyses, participants were classified according to regular participation in moderate-to-vigorous leisure-time physical activity. Participants not reporting regular moderate-to-vigorous leisure-time physical activity were classified as physically inactive [52].

Adherence to Mediterranean dietary patterns was estimated using nutritional habit questionnaires focused on regular consumption of fruits, vegetables, legumes, olive oil, fish, and ultra-processed foods. For the present analyses, Mediterranean diet adherence was categorized as high versus low adherence according to the dietary assessment used in the occupational health examination [53]. Alcohol consumption was categorized for the present analyses as regular consumption versus no habitual consumption.

### 2.6. Definition of Metabolic Dysfunction Phenotypes

To evaluate adiposity–metabolic discordance among adults with normal BMI, participants were stratified according to visceral adiposity burden and metabolic abnormalities.

Metabolic dysfunction was defined by the presence of at least one of the following conditions:

- elevated fasting glucose,
- hypertension,
- hypertriglyceridemia,
- reduced HDL cholesterol,
- or increased visceral adiposity indices.

This multidimensional approach was selected because isolated BMI classification may underestimate early cardiometabolic deterioration in apparently normal-weight adults [54,55].

Participants were subsequently categorized into metabolically favorable and metabolically unfavorable phenotypes to explore differences in residual atherogenic risk.

### 2.7. Statistical Analysis

Continuous variables are presented as mean ± standard deviation, whereas categorical variables are expressed as absolute frequencies and percentages.

Comparisons between groups were performed using Student’s *t* test or analysis of variance (ANOVA) for continuous variables and chi-square tests for categorical variables. Correlations between anthropometric, metabolic, and lipid-related variables were assessed using Pearson correlation coefficients.

Multivariable logistic regression analyses were performed to identify factors independently associated with elevated TG/HDL-c. To minimize collinearity among adiposity-related measures, BMI, waist-to-height ratio (WtHR), and METS-VF were not entered simultaneously. Instead, three separate models were constructed, each including one adiposity-related measure (BMI, WtHR, or METS-VF) together with the same set of covariates.

Statistical significance was established at *p* < 0.05. All analyses were conducted using IBM SPSS Statistics software version 30.0 (IBM Corp., Armonk, NY, USA).

## 3. Results

### 3.1. Study Population

A total of 166,008 normal-weight adults were included. Mean age was 36.99 ± 9.83 years, and 49.4% were men. Mean BMI was 22.45 ± 1.67 kg/m^2^. Despite normal BMI values, relevant cardiometabolic heterogeneity was observed: 11.8% had increased central adiposity, 8.0% had hypertriglyceridemia, 11.4% had low HDL cholesterol, 7.3% had impaired fasting glucose, and 17.9% had elevated blood pressure.

The baseline anthropometric, metabolic, lipid, and lifestyle characteristics of the study population are summarized in Table 1.

Although all participants were classified as normal weight according to BMI criteria, a substantial proportion already exhibited unfavorable cardiometabolic characteristics, particularly increased central adiposity, an elevated TG/HDL-c ratio, impaired fasting glucose, and elevated blood pressure, suggesting the presence of hidden metabolic dysfunction within this apparently low-risk population.

Overall, 8.0% of participants showed an elevated TG/HDL-c ratio ≥ 3.0, while 1.7% fulfilled the stricter definition of classic atherogenic dyslipidemia based on concomitant hypertriglyceridemia and low HDL cholesterol.

**Figure 2:**
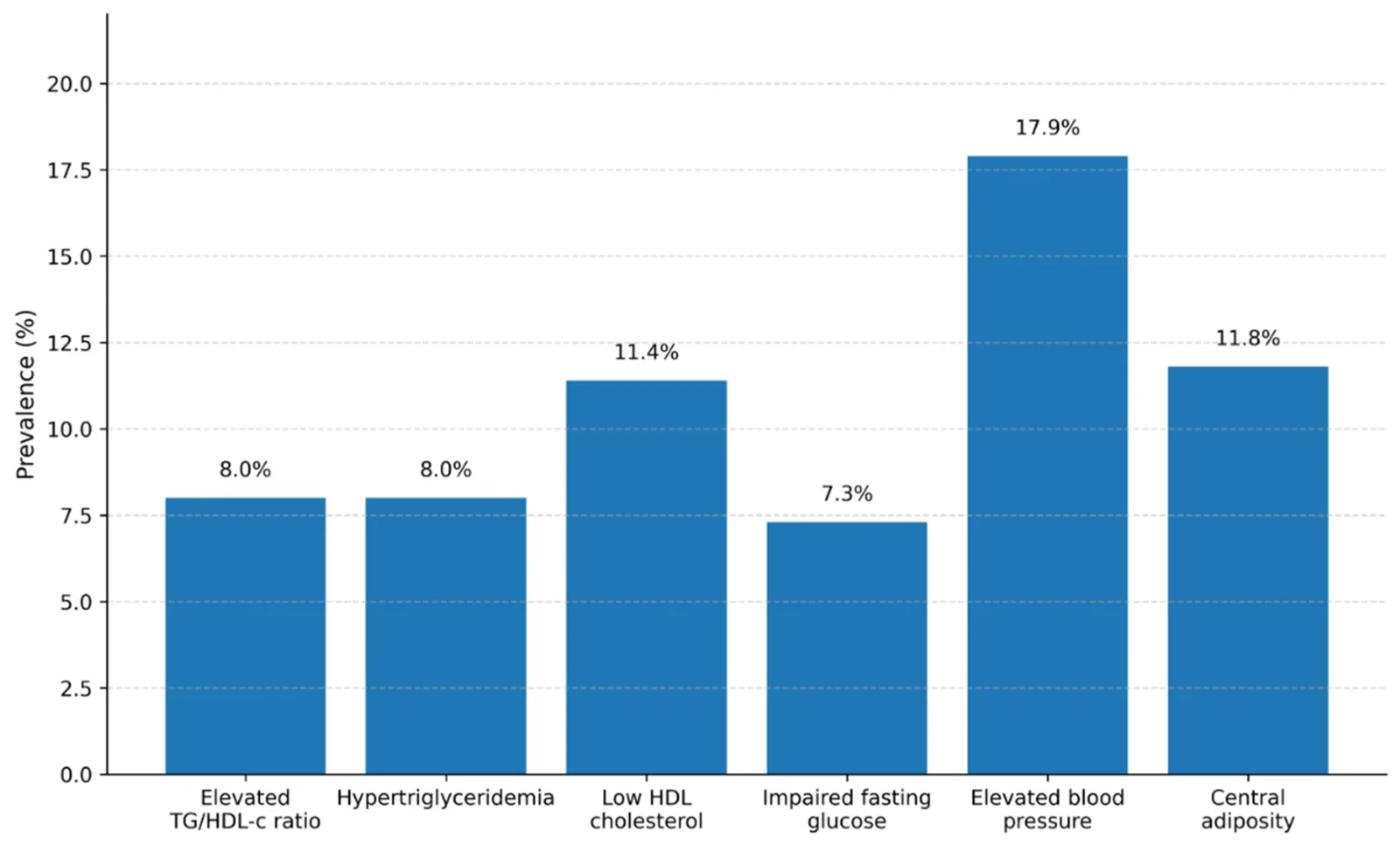
Prevalence of residual atherogenic lipid abnormalities in normal-weight adults.

The prevalence of residual atherogenic lipid abnormalities and other hidden cardiometabolic alterations among normal-weight adults is presented in Figure 2.

Prevalence of residual atherogenic lipid abnormalities and cardiometabolic alterations in normal-weight adults. An elevated TG/HDL-c ratio was defined as a value ≥3.0. Central adiposity was defined as waist-to-height ratio ≥ 0.50. HDL-c: high-density lipoprotein cholesterol; TG/HDL-c: triglyceride-to-HDL cholesterol ratio.

Despite all participants being classified as normal weight according to conventional BMI criteria, a considerable proportion already exhibited clinically relevant hidden cardiometabolic abnormalities, particularly elevated blood pressure, central adiposity, low HDL cholesterol, and residual atherogenic dyslipidemia, supporting the existence of substantial metabolic heterogeneity within apparently low-risk individuals.

### 3.2. Lipid and Cardiometabolic Profile According to Residual Atherogenic Dyslipidemia

Participants with an elevated TG/HDL-c ratio showed a clearly adverse lipid and cardiometabolic profile despite remaining within the normal BMI range. Compared with participants without an elevated TG/HDL-c ratio, they were older, had higher BMI, waist circumference, WtHR, METS-VF, blood pressure, fasting glucose, total cholesterol, LDL cholesterol, triglycerides, non-HDL cholesterol, and TyG index, together with lower HDL cholesterol.

Mean triglyceride concentrations were 211.49 ± 87.54 mg/dL in participants with an elevated TG/HDL-c ratio compared with 77.45 ± 28.53 mg/dL in those without it. HDL cholesterol was markedly lower in the elevated TG/HDL-c group (47.26 ± 7.56 mg/dL vs. 54.55 ± 6.92 mg/dL). Non-HDL cholesterol was also substantially higher (170.11 ± 43.74 mg/dL vs. 130.40 ± 36.99 mg/dL).

The anthropometric, metabolic, vascular, and lipid-related characteristics according to residual atherogenic dyslipidemia status are summarized in Table 2.

Participants with an elevated TG/HDL-c ratio exhibited a markedly adverse cardiometabolic profile despite remaining within the normal BMI range. The most pronounced differences were observed for triglycerides, HDL cholesterol, TyG index, central adiposity, blood pressure, and non-HDL cholesterol, indicating that residual atherogenic dyslipidemia identifies a subgroup of apparently normal-weight adults with substantial hidden metabolic and vascular risk.

The strongest differences were observed for triglycerides, TG/HDL-c ratio, HDL cholesterol, non-HDL cholesterol, TC/HDL-c ratio, and TyG index, indicating that residual atherogenic dyslipidemia identifies a lipid-metabolic phenotype not captured by BMI alone.

**Figure 3:**
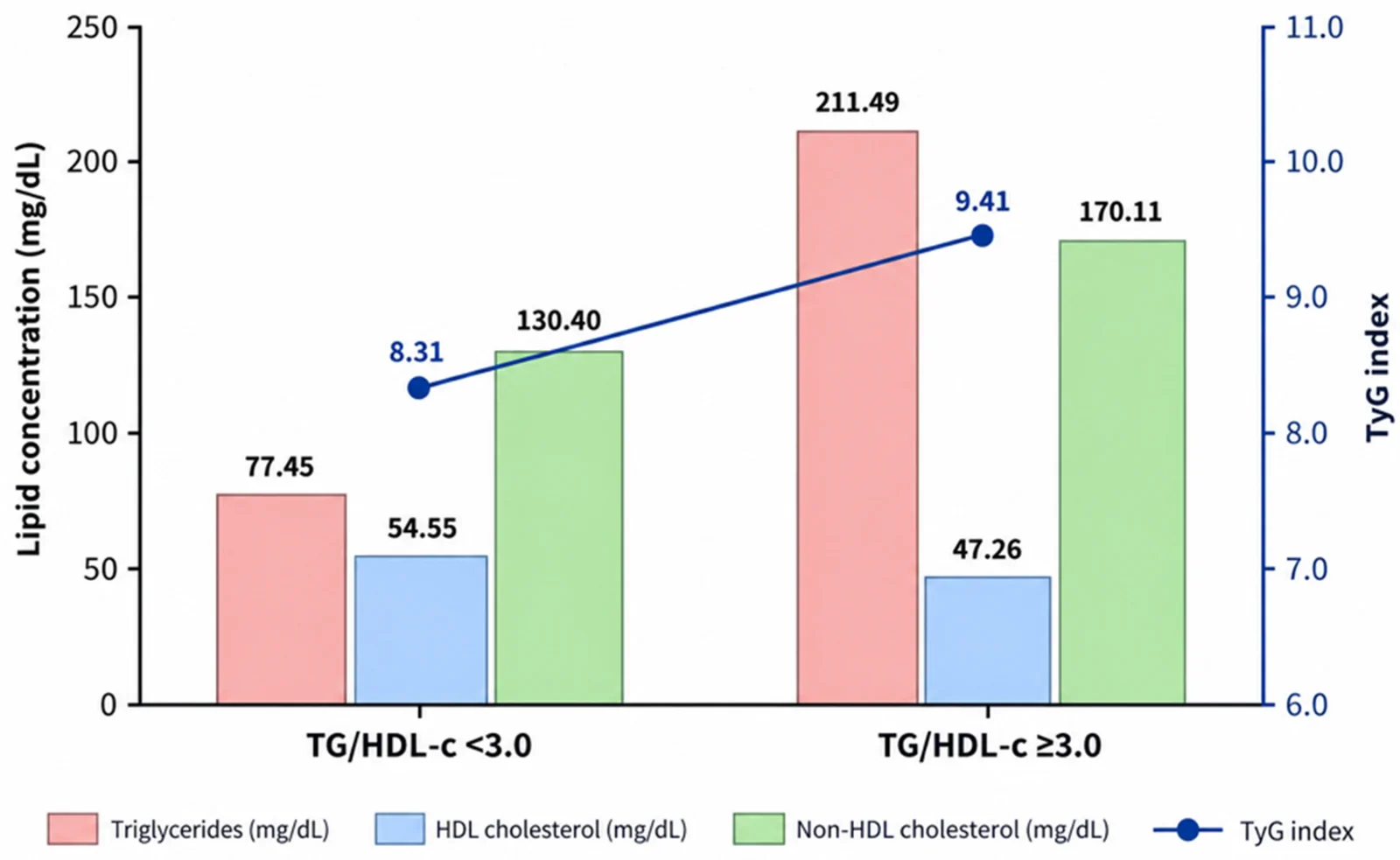
Lipid and metabolic profile according to residual atherogenic dyslipidemia status. Triglycerides, HDL cholesterol, and non-HDL cholesterol are represented on the primary Y-axis (left; mg/dL), whereas the TyG index is represented on the secondary Y-axis (right). Participants with TG/HDL-c ratio ≥ 3.0 showed higher triglycerides, non-HDL cholesterol, and TyG index values and lower HDL cholesterol concentrations than participants with TG/HDL-c ratio < 3.0. HDL-c, high-density lipoprotein cholesterol; TG/HDL-c, triglyceride-to-high-density lipoprotein cholesterol ratio; TyG, triglyceride-glucose index.

Figure 3 illustrates the differences in the lipid-metabolic profile between participants with and without residual atherogenic dyslipidemia.

The figure demonstrates the profound metabolic divergence observed despite normal BMI values in both groups. Individuals with an elevated TG/HDL-c ratio exhibited a characteristic atherogenic pattern defined by severe hypertriglyceridemia, reduced HDL cholesterol, increased non-HDL cholesterol burden, and higher insulin resistance-related indices, supporting the presence of substantial residual cardiometabolic risk beyond conventional anthropometric classification.

### 3.3. Residual Atherogenic Dyslipidemia According to Visceral Adiposity Burden

A progressive worsening of lipid and cardiometabolic parameters was observed across increasing METS-VF quartiles. Participants in the highest METS-VF quartile were older and had higher BMI, waist circumference, WtHR, triglycerides, TG/HDL-c ratio, non-HDL cholesterol, fasting glucose, and blood pressure, with lower HDL cholesterol.

The prevalence of an elevated TG/HDL-c ratio increased progressively from 2.7% in Q1 to 15.8% in Q4. Classic atherogenic dyslipidemia also increased from 1.0% in Q1 to 3.1% in Q4. Hypertriglyceridemia increased from 2.9% to 15.4%, while impaired fasting glucose increased from 3.4% to 12.3%.

The distribution of anthropometric, metabolic, vascular, and lipid-related variables across increasing METS-VF quartiles is shown in Table 3.

A clear dose–response relationship was observed across increasing METS-VF quartiles. Participants with greater visceral adiposity burden showed progressively worse anthropometric, hemodynamic, metabolic, and lipid-related profiles, including higher triglycerides, TG/HDL-c ratio, TyG index, fasting glucose, and blood pressure values together with lower HDL cholesterol concentrations, reinforcing the close association between visceral adiposity and residual atherogenic cardiometabolic risk even within the normal BMI range.

This gradient suggests that visceral adiposity-related metabolic dysfunction is closely associated with residual atherogenic lipid risk even among individuals with normal BMI.

**Figure 4:**
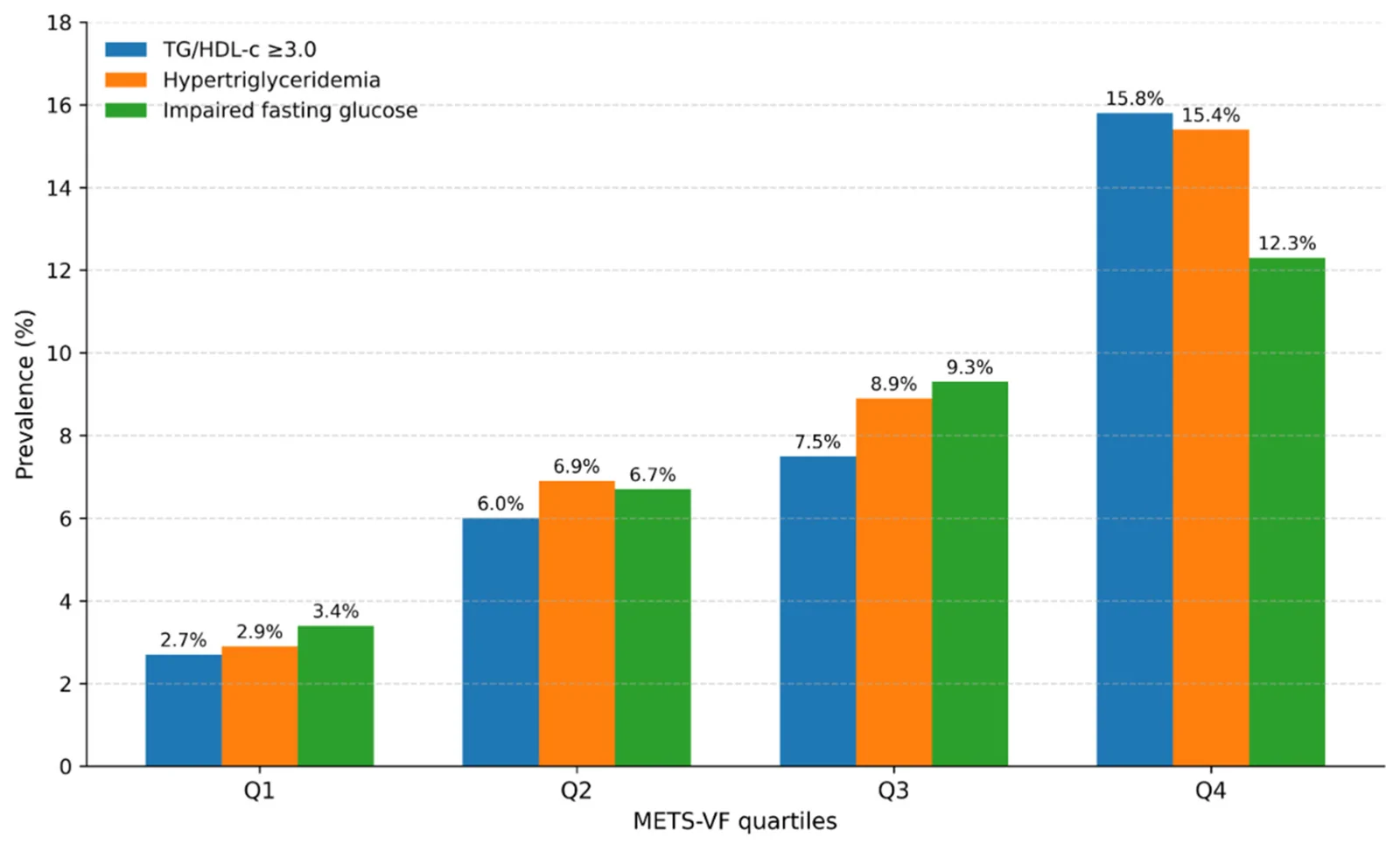
Dose–response relationship between METS-VF quartiles and residual atherogenic dyslipidemia. Prevalence of residual atherogenic dyslipidemia, hypertriglyceridemia, and impaired fasting glucose according to METS-VF quartiles. A progressive increase in cardiometabolic abnormalities was observed across increasing visceral adiposity burden. METS-VF: Metabolic Score for Visceral Fat; TG/HDL-c: triglyceride-to-HDL cholesterol ratio.

The progressive relationship between visceral adiposity burden and residual atherogenic cardiometabolic abnormalities is illustrated in Figure 4.

A marked dose–response pattern was identified across increasing METS-VF quartiles. Participants in the highest visceral adiposity quartile exhibited substantially greater prevalence of an elevated TG/HDL-c ratio, hypertriglyceridemia, and impaired fasting glucose compared with those in the lowest quartile, supporting the close biological relationship between visceral fat accumulation and residual atherogenic-metabolic dysfunction even among individuals with normal BMI values.

### 3.4. Central Adiposity and Residual Atherogenic Lipid Risk

Participants with WtHR ≥ 0.50 showed a less favorable lipid profile than those with normal WtHR. Mean triglycerides were higher in participants with central adiposity (98.55 ± 58.89 mg/dL vs. 86.72 ± 50.45 mg/dL), while HDL cholesterol was lower (52.75 ± 7.80 mg/dL vs. 54.13 ± 7.15 mg/dL). The TG/HDL-c ratio was also higher in the central adiposity group.

The prevalence of an elevated TG/HDL-c ratio was 12.8% among participants with central adiposity compared with 7.3% among those without central adiposity. Classic atherogenic dyslipidemia was also more frequent in the central adiposity group (2.5% vs. 1.7%).

The relationship between central adiposity and residual atherogenic lipid abnormalities among normal-weight adults is presented in Table 4.

Normal-weight adults with central adiposity exhibited a substantially less favorable cardiometabolic profile than those without increased waist-related adiposity. In addition to higher triglycerides, non-HDL cholesterol, TG/HDL-c ratio, and TyG index values, these individuals also showed greater prevalence of impaired fasting glucose and elevated blood pressure, reinforcing the importance of waist-related adiposity assessment for identifying hidden residual atherogenic risk beyond BMI classification alone.

These findings indicate that waist-related adiposity identifies a subgroup of normal-weight adults with greater hidden lipid-related cardiometabolic burden.

### 3.5. Sex-Specific Differences in Residual Atherogenic Dyslipidemia

Marked sex differences were observed. Men had higher BMI, triglycerides, TG/HDL-c ratio, and prevalence of central adiposity, whereas women had higher HDL cholesterol but also a higher prevalence of low HDL according to sex-specific thresholds.

An elevated TG/HDL-c ratio was present in 12.2% of men and 3.9% of women. Hypertriglyceridemia was also more frequent in men (12.5% vs. 3.7%). Conversely, low HDL cholesterol was more frequent in women using sex-specific criteria (19.7% vs. 3.0%). Classic atherogenic dyslipidemia was slightly more frequent in men than women (2.0% vs. 1.5%).

Sex-specific differences in anthropometric, lipid, metabolic, and vascular parameters are summarized in Table 5.

Marked sex-related differences were identified across most anthropometric, metabolic, vascular, and lipid parameters. Men exhibited substantially higher visceral adiposity burden, triglycerides, TG/HDL-c ratio, non-HDL cholesterol, blood pressure, and prevalence of residual atherogenic dyslipidemia, whereas women showed higher HDL cholesterol concentrations but also a greater prevalence of low HDL cholesterol according to sex-specific criteria. These findings suggest that sex-related biological differences may significantly modulate hidden cardiometabolic and atherogenic risk among normal-weight adults.

**Figure 5:**
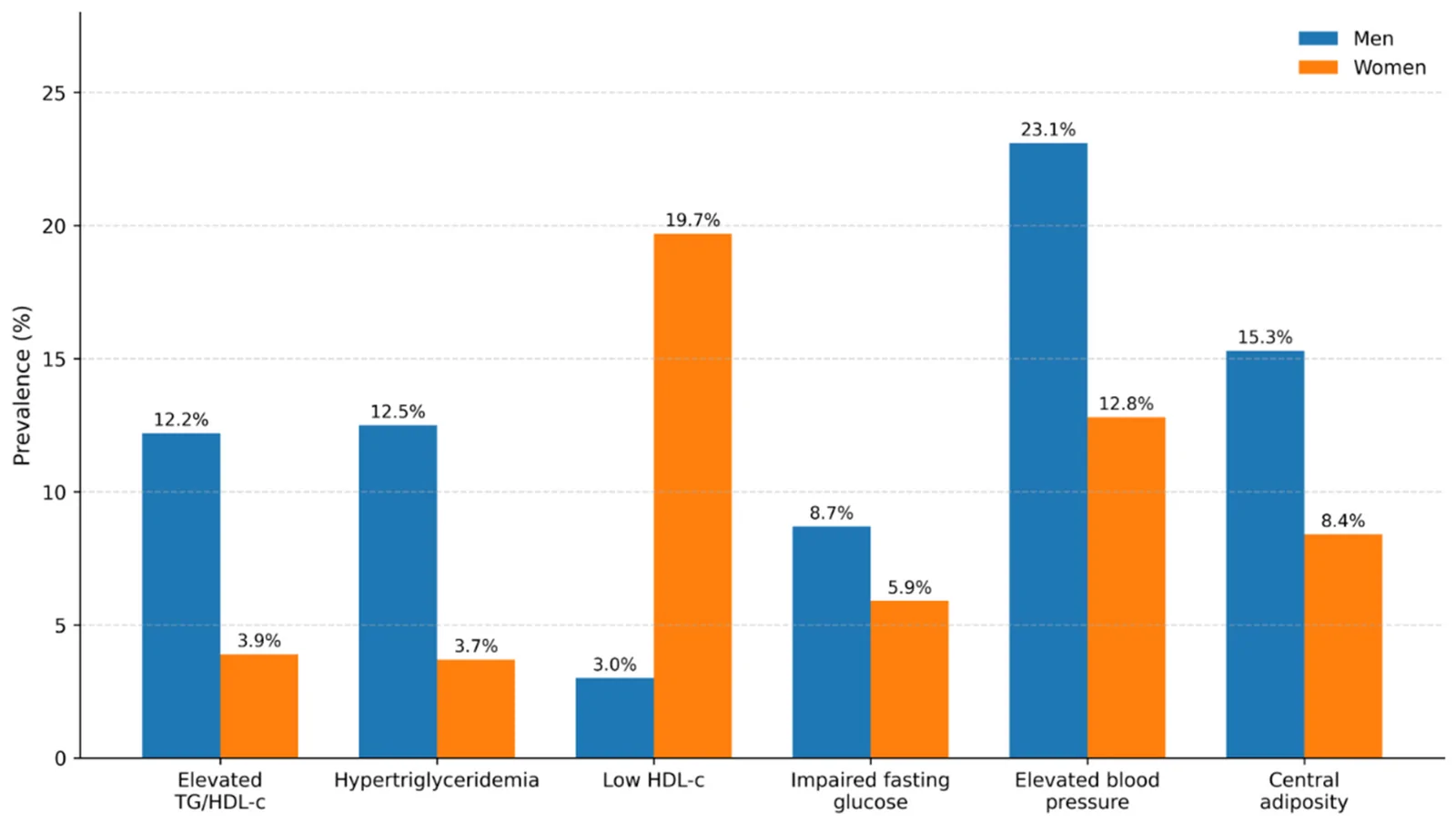
Sex differences in residual atherogenic lipid abnormalities among normal-weight adults. Sex differences in residual atherogenic lipid abnormalities and cardiometabolic alterations among normal-weight adults. Men showed a higher prevalence of an elevated TG/HDL-c ratio, hypertriglyceridemia, elevated blood pressure, impaired fasting glucose, and central adiposity, whereas women exhibited a higher prevalence of low HDL cholesterol according to sex-specific thresholds. HDL-c: high-density lipoprotein cholesterol; TG/HDL-c: triglyceride-to-high-density lipoprotein cholesterol ratio.

Sex-related differences in residual atherogenic lipid abnormalities and hidden cardiometabolic risk are illustrated in Figure 5.

The figure highlights the marked sexual dimorphism underlying residual cardiometabolic risk despite normal BMI values. Men displayed a substantially more adverse visceral adiposity and atherogenic lipid profile, whereas women preserved higher HDL cholesterol concentrations overall but showed a greater frequency of low HDL cholesterol when sex-adjusted cut-off values were applied. These findings reinforce the relevance of sex-specific approaches in the identification and interpretation of hidden metabolic dysfunction among apparently healthy normal-weight individuals.

### 3.6. Lifestyle-Related Profile of Residual Atherogenic Dyslipidemia

Residual atherogenic dyslipidemia was strongly associated with unfavorable lifestyle-related characteristics. An elevated TG/HDL-c ratio was present in 35.3% of physically inactive participants compared with only 0.4% of physically active participants. It was also more frequent among participants with poor dietary adherence than among those with favorable dietary adherence (26.7% vs. 0.8%). Current smokers showed a higher prevalence of an elevated TG/HDL-c ratio than non-smokers (11.0% vs. 6.0%).

These differences indicate that lifestyle-related factors are closely linked to hidden atherogenic lipid abnormalities even in the absence of overweight or obesity.

The prevalence of residual atherogenic dyslipidemia according to lifestyle-related variables is summarized in Table 6.

Residual atherogenic dyslipidemia was consistently associated with unfavorable lifestyle-related behaviors. Physically inactive participants, participants with low adherence to the Mediterranean diet, current smokers, and those reporting regular alcohol consumption exhibited substantially higher prevalence of an elevated TG/HDL-c ratio and hypertriglyceridemia. These findings indicate that unfavorable lifestyle-related characteristics are strongly associated with hidden atherogenic-metabolic dysfunction even among adults with apparently normal body weight.

### 3.7. Multivariable Analysis

In multivariable logistic regression analyses using separate adiposity measures, BMI and METS-VF remained independently associated with elevated TG/HDL-c after adjustment for demographic, metabolic, and lifestyle-related factors. In contrast, WtHR ≥ 0.50 was not independently associated with elevated TG/HDL-c in the corresponding adjusted model (OR 0.96; 95% CI 0.91–1.03; *p* = 0.250). The associations of male sex, impaired fasting glucose, elevated blood pressure, physical inactivity, poor dietary adherence, and current smoking remained broadly consistent across the three models.

The independent variables associated with elevated TG/HDL-c were evaluated using three separate multivariable logistic regression models according to the adiposity measure included, as shown in Table 7.

Across the three multivariable models, male sex, impaired fasting glucose, elevated blood pressure, physical inactivity, low Mediterranean diet adherence, and current smoking remained independently associated with elevated TG/HDL-c. Regarding adiposity-related measures, BMI was independently associated with elevated TG/HDL-c in Model 1 (OR 1.13; 95% CI 1.11–1.15), whereas WtHR ≥ 0.50 was not independently associated in Model 2 (OR 0.96; 95% CI 0.91–1.03). METS-VF remained independently associated with elevated TG/HDL-c in Model 3 (OR 1.30; 95% CI 1.24–1.37). The association observed for physical inactivity remained unusually large and consistent across all three models and should therefore be interpreted with caution.

### 3.8. Integrated Summary of Findings

Overall, these results demonstrate that residual atherogenic dyslipidemia is present in a clinically relevant proportion of normal-weight adults and is strongly associated with visceral adiposity burden, metabolic dysfunction, male sex, and unfavorable lifestyle-related behaviors. Although all participants had BMI values within the normal range, individuals with an elevated TG/HDL-c ratio showed a markedly adverse lipid-metabolic phenotype characterized by higher triglycerides, lower HDL cholesterol, higher non-HDL cholesterol, increased TyG index, higher METS-VF, and greater prevalence of impaired fasting glucose and elevated blood pressure.

These findings support the concept that normal BMI does not exclude clinically meaningful atherogenic lipid risk and reinforce the need for lipid-metabolic assessment beyond conventional weight classification.

## 4. Discussion

### 4.1. Principal Findings

The present study demonstrates that a clinically relevant proportion of adults classified as normal weight according to conventional BMI criteria already exhibit substantial residual atherogenic dyslipidemia and hidden cardiometabolic dysfunction. Despite apparently normal body weight, individuals with an elevated TG/HDL-c ratio showed markedly adverse lipid, metabolic, vascular, and adiposity-related profiles characterized by higher triglycerides, non-HDL cholesterol, blood pressure, fasting glucose, visceral adiposity burden, and insulin resistance-related indices. Importantly, these abnormalities were accompanied by differences in adiposity-related measures, while the multivariable analyses showed an independent association for METS-VF but not for WtHR ≥ 0.50 after adjustment.

These findings reinforce the growing body of evidence indicating that adiposity distribution and adipose tissue dysfunction may be more relevant determinants of cardiometabolic risk than total body weight alone [56,57]. In recent years, increasing attention has been directed toward the biological heterogeneity of obesity and toward the recognition that metabolically unfavorable phenotypes may occur in apparently lean individuals [58,59]. Our results extend this concept by specifically demonstrating that residual atherogenic dyslipidemia is highly prevalent even among adults who would traditionally be considered low-risk according to standard anthropometric criteria.

The magnitude of the observed metabolic abnormalities is particularly relevant from a preventive cardiology perspective. Participants with an elevated TG/HDL-c ratio exhibited substantial increases in central adiposity, impaired fasting glucose, elevated blood pressure, and non-HDL cholesterol despite remaining within the normal BMI range. These alterations are closely linked to early insulin resistance, endothelial dysfunction, remnant lipoprotein accumulation, and accelerated atherosclerotic progression [60,61]. Consequently, the present data support the notion that residual cardiometabolic risk may already be established long before overt obesity or clinically manifest cardiovascular disease become apparent.

Our findings are also consistent with current pathophysiological models emphasizing the central role of visceral adipose tissue in cardiometabolic deterioration [62]. Visceral fat accumulation promotes increased lipolytic activity, excessive free fatty acid flux to the liver, hepatic insulin resistance, chronic low-grade inflammation, and altered lipoprotein metabolism, thereby favoring the development of hypertriglyceridemia, reduced HDL cholesterol concentrations, and increased remnant lipoproteins [63,64]. In this context, the independent association observed for METS-VF, together with the unadjusted differences observed according to WtHR categories, further supports the relevance of adiposity distribution in residual atherogenic dyslipidemia.

Importantly, these findings challenge the widespread assumption that normal BMI necessarily reflects favorable metabolic health. The progressive increase in atherogenic abnormalities across increasing visceral adiposity quartiles indicates a close cross-sectional association between visceral adiposity burden and metabolic dysfunction even within the normal BMI range. This observation supports previous evidence indicating that limited peripheral adipose tissue expandability and ectopic fat deposition may contribute substantially to early insulin resistance and lipid-related cardiometabolic dysfunction independently of BMI [65,66].

### 4.2. Residual Atherogenic Dyslipidemia and Hidden Cardiometabolic Risk

Using the primary operational definition of TG/HDL-c ≥ 3.0, residual atherogenic dyslipidemia was present in 8.0% of the study population. In contrast, only 1.7% fulfilled the stricter definition of classic atherogenic dyslipidemia based on concomitant hypertriglyceridemia and low HDL-c. This difference reflects the broader lipid-metabolic phenotype captured by the TG/HDL-c ratio compared with the conventional definition requiring simultaneous abnormalities in both lipid components. These findings suggest that traditional weight-based classification may substantially underestimate hidden cardiovascular and metabolic vulnerability.

The TG/HDL-c ratio has emerged as one of the most robust lipid-derived markers of insulin resistance, remnant lipoprotein burden, endothelial dysfunction, and residual cardiovascular risk [67,68]. Elevated values have been consistently associated with increased prevalence of metabolic syndrome, coronary artery calcification, non-alcoholic fatty liver disease, and incident cardiovascular disease even in individuals without overt obesity [69,70]. Our findings reinforce this evidence and demonstrate that these abnormalities are already detectable in apparently healthy normal-weight adults.

Particularly noteworthy was the marked deterioration in TyG index, non-HDL cholesterol, TC/HDL-c ratio, and LDL-c/HDL-c ratio among participants with elevated TG/HDL-c values. These alterations support the existence of a broad atherogenic-metabolic phenotype characterized not only by quantitative lipid abnormalities but also by profound metabolic dysregulation. Previous investigations have demonstrated that triglyceride-rich lipoproteins and remnant cholesterol particles contribute significantly to residual cardiovascular risk independently of LDL cholesterol concentrations [71,72]. Consequently, reliance exclusively on conventional lipid parameters may fail to identify individuals at increased cardiometabolic risk despite apparently acceptable LDL cholesterol values.

The concept of residual atherogenic risk has gained considerable relevance in contemporary preventive cardiology because cardiovascular events continue to occur even among individuals with relatively controlled traditional risk factors [73]. Increasing evidence suggests that insulin resistance, visceral adiposity, chronic inflammation, remnant lipoproteins, and adipose tissue dysfunction play fundamental roles in this persistent risk burden [74]. The present results strongly support this paradigm and indicate that residual cardiometabolic dysfunction may already be present at early stages among normal-weight adults.

Furthermore, our findings reinforce the limitations of BMI as an isolated screening tool for cardiometabolic risk stratification. Although BMI remains useful at the population level, it does not adequately capture visceral adiposity, ectopic fat deposition, or adipose tissue functionality [75]. As a result, substantial metabolic heterogeneity may remain clinically masked within individuals classified as normal weight.

### 4.3. Visceral Adiposity as a Central Mechanism

The progressive worsening of cardiometabolic variables across increasing METS-VF quartiles represents one of the strongest findings of the present study. Participants in the highest visceral adiposity quartile showed substantially higher prevalence of an elevated TG/HDL-c ratio, impaired fasting glucose, hypertriglyceridemia, elevated blood pressure, and central adiposity compared with those in the lowest quartile, despite all participants maintaining BMI values within the normal range.

These observations are biologically plausible because visceral adipose tissue differs substantially from subcutaneous fat in its endocrine, inflammatory, and metabolic activity [76,77]. Visceral fat exhibits greater release of free fatty acids, increased secretion of proinflammatory cytokines, impaired adipokine regulation, and greater hepatic portal drainage, all of which contribute directly to insulin resistance and atherogenic dyslipidemia [78]. Experimental and clinical studies have consistently demonstrated that visceral adiposity is more strongly associated with cardiovascular disease, metabolic syndrome, and mortality than total adiposity itself [79,80].

The present findings showed different patterns for the two adiposity-related measures: METS-VF remained independently associated with elevated TG/HDL-c in the adjusted analysis, whereas WtHR ≥ 0.50 did not retain an independent association after adjustment. Several recent studies have shown that WtHR may outperform BMI in predicting diabetes, hypertension, fatty liver disease, and cardiovascular events [81,82]. Likewise, METS-VF has emerged as a promising surrogate marker of visceral adiposity integrating anthropometric and metabolic information into a single clinically applicable index [83,84].

Our findings also align with current concepts regarding adipose tissue expandability and adipose dysfunction. According to these models, individuals with limited subcutaneous adipose tissue storage capacity may develop earlier ectopic fat deposition and metabolic deterioration even without substantial increases in total body weight [85]. This mechanism may partially explain why some apparently lean individuals already present pronounced cardiometabolic abnormalities despite remaining within normal BMI ranges.

### 4.4. Lifestyle Factors and Metabolic Vulnerability

Lifestyle-related variables showed remarkably strong associations with residual atherogenic dyslipidemia in the present study. Physical inactivity, smoking, poor Mediterranean diet adherence, and regular alcohol consumption were all associated with a markedly higher prevalence of hidden atherogenic-metabolic abnormalities.

The unusually large association observed for physical inactivity deserves cautious interpretation. Following verification of the physical activity coding and repetition of the regression analysis, no coding or software error was identified, and the strong association was already evident in the unadjusted analysis. Thus, although the association was reproducible in the present dataset, its magnitude should not be interpreted as representing a biological effect of comparable size. Physical inactivity has been associated with insulin resistance, impaired skeletal muscle glucose utilization, altered triglyceride metabolism, endothelial dysfunction, and visceral fat accumulation [86,87,88]. Accordingly, the present findings support an association between physical inactivity and an adverse metabolic profile, while the magnitude of the estimated OR should be interpreted cautiously.

Similarly, Mediterranean dietary adherence appeared strongly protective against residual atherogenic dyslipidemia. Mediterranean dietary patterns are characterized by high intake of fruits, vegetables, legumes, olive oil, fish, and unsaturated fatty acids, all of which exert beneficial effects on lipid metabolism, oxidative stress, endothelial function, and systemic inflammation [89,90]. Clinical trials and large cohort studies have demonstrated significant reductions in cardiovascular events and metabolic disease associated with Mediterranean dietary patterns independently of body weight changes [91].

Smoking also remained independently associated with an elevated TG/HDL-c ratio after multivariable adjustment. Tobacco exposure promotes oxidative stress, endothelial injury, sympathetic activation, altered lipid metabolism, and preferential visceral fat accumulation [92,93]. Several Mendelian randomization studies have further suggested potential causal relationships between smoking and abdominal adiposity accumulation [94]. These mechanisms likely contribute to the adverse lipid-metabolic profile observed among smokers in the present cohort.

Collectively, these findings highlight that cardiometabolic vulnerability among normal-weight adults is not determined exclusively by anthropometric status but also by complex behavioral and metabolic interactions that may remain clinically silent for prolonged periods.

### 4.5. Sex-Related Differences in Residual Cardiometabolic Risk

The marked sexual dimorphism identified in the present investigation is consistent with previous evidence demonstrating important biological differences in adiposity distribution, lipid metabolism, and cardiovascular risk between men and women [95]. Men exhibited substantially higher visceral adiposity burden, triglycerides, non-HDL cholesterol, TG/HDL-c ratio, blood pressure, and prevalence of residual atherogenic dyslipidemia, whereas women showed higher HDL cholesterol concentrations overall.

These differences may be partially explained by sex-specific hormonal regulation of adipose tissue distribution and lipid metabolism. Men tend to accumulate greater amounts of visceral adipose tissue, whereas women more frequently exhibit peripheral and subcutaneous fat distribution patterns during reproductive years [96]. Visceral adiposity is metabolically more active and strongly associated with insulin resistance, remnant lipoprotein accumulation, and endothelial dysfunction, thereby contributing to greater atherogenic risk among men [97].

Nevertheless, women demonstrated a higher prevalence of low HDL cholesterol according to sex-specific criteria. This finding suggests that hidden metabolic alterations may also remain clinically relevant among normal-weight women despite apparently favorable overall lipid profiles. Previous studies have demonstrated that menopause, hormonal changes, and alterations in adipose tissue distribution may progressively increase cardiometabolic risk among women even in the absence of overt obesity [98].

These observations reinforce the importance of incorporating sex-specific approaches into cardiometabolic risk assessment and support the need for more individualized metabolic evaluation beyond BMI classification alone.

### 4.6. Clinical Implications

The present findings may have important implications for preventive cardiology and public health strategies. Current obesity screening approaches remain predominantly focused on BMI-based classification despite growing evidence demonstrating its limited ability to identify hidden metabolic dysfunction [99]. Our data suggest that a substantial subgroup of adults currently considered low-risk may already exhibit clinically meaningful atherogenic and metabolic abnormalities.

Assessment of waist-related, visceral adiposity, and lipid-derived cardiometabolic indices may provide complementary information on the cardiometabolic profile of apparently healthy normal-weight individuals.

From a preventive perspective, recognition of metabolic abnormalities among normal-weight individuals may contribute to a more comprehensive cross-sectional cardiometabolic assessment. These considerations are especially relevant given the growing global burden of obesity-related diseases and the increasing recognition of metabolically adverse phenotypes outside traditional obesity definitions [100].

### 4.7. Strengths and Limitations

The major strengths of the present study include the very large sample size, the inclusion of both sexes, the standardized occupational health assessment procedures, and the simultaneous evaluation of anthropometric, metabolic, vascular, and lifestyle-related variables. Additionally, the use of multiple adiposity- and lipid-related indices allowed a more comprehensive characterization of hidden cardiometabolic dysfunction among normal-weight adults.

Several limitations should nevertheless be acknowledged. First, the cross-sectional design prevents establishing temporal or causal relationships between visceral adiposity, lifestyle factors, and residual atherogenic dyslipidemia; therefore, the observed associations should not be interpreted as evidence of future risk prediction. Second, lifestyle variables were partially self-reported and therefore susceptible to recall bias. Third, the study population consisted exclusively of actively employed adults, potentially limiting generalizability to older or clinically ill populations. Finally, direct imaging-based quantification of visceral adipose tissue was not available.

A further limitation concerns the unusually large association observed between physical inactivity and elevated TG/HDL-c. The marked prevalence contrast between physically inactive and active participants (35.3% vs. 0.4%) resulted in adjusted odds ratios exceeding 100 across the multivariable models. Although the coding and analytical procedures were verified, the magnitude of this estimate may partly reflect the dichotomous classification of physical activity, residual or unmeasured confounding, and sparse-data or quasi-separation-related behavior due to the very low outcome prevalence in the physically active reference group. Therefore, this odds ratio should not be interpreted as a conventional quantitative effect-size estimate or as evidence of a causal association of this magnitude, but rather as indicating a very strong association within the specific operational definitions and structure of the present dataset.

In addition, ApoB and LDL particle number (LDL-P), which provide a more direct assessment of circulating atherogenic particle burden, were not available in the occupational health database and therefore could not be evaluated. This limitation is particularly relevant because increased atherogenic particle burden may occur despite apparently normal LDL-C concentrations. Consequently, the lipid-derived indices evaluated in the present study should not be interpreted as substitutes for direct ApoB or LDL-P measurement.

Despite these limitations, the consistency of the observed associations and the large sample size strongly support the robustness of the findings.

## 5. Conclusions

Residual atherogenic dyslipidemia is highly prevalent among adults with normal BMI values and is strongly associated with visceral adiposity burden, metabolic dysfunction, male sex, physical inactivity, smoking, and poor dietary adherence. Waist-related and lipid-derived indices provided complementary information beyond BMI for the cross-sectional characterization of hidden cardiometabolic risk.

These findings reinforce the concept that normal body weight does not necessarily indicate metabolic health and highlight the potential value of considering visceral adiposity and lipid-metabolic characteristics alongside BMI when characterizing cardiometabolic health.

## Figures and Tables

**Table 1:** Baseline anthropometric, metabolic, lipid, and lifestyle characteristics of the study population.

Variable	Total Population (n = 166,008)
Age (years)	36.99 ± 9.83
Male sex, n (%)	81,996 (49.4)
BMI (kg/m^2^)	22.45 ± 1.67
Waist circumference (cm)	78.84 ± 8.91
Waist-to-height ratio	0.47 ± 0.05
METS-VF	5.32 ± 0.71
Systolic blood pressure (mmHg)	118.72 ± 13.64
Diastolic blood pressure (mmHg)	74.69 ± 9.18
Fasting glucose (mg/dL)	91.43 ± 11.72
Total cholesterol (mg/dL)	187.62 ± 35.94
LDL cholesterol (mg/dL)	114.31 ± 31.67
HDL cholesterol (mg/dL)	53.99 ± 7.31
Triglycerides (mg/dL)	88.56 ± 52.41
Non-HDL cholesterol (mg/dL)	133.63 ± 37.21
TG/HDL-c ratio	1.79 ± 1.26
TC/HDL-c ratio	3.54 ± 0.91
Elevated TG/HDL-c ratio ≥ 3.0, n (%)	13,281 (8.0)
Hypertriglyceridemia, n (%)	13,278 (8.0)
Low HDL cholesterol, n (%)	18,925 (11.4)
Impaired fasting glucose, n (%)	12,118 (7.3)
Elevated blood pressure, n (%)	29,715 (17.9)
Central adiposity (WtHR ≥ 0.50), n (%)	19,589 (11.8)
Current smokers, n (%)	56,774 (34.2)
Physical inactivity, n (%)	58,934 (35.5)
Low Mediterranean diet adherence, n (%)	44,156 (26.6)

Data are presented as mean ± standard deviation or absolute frequency and percentage. BMI: body mass index; HDL-c: high-density lipoprotein cholesterol; LDL-c: low-density lipoprotein cholesterol; METS-VF: Metabolic Score for Visceral Fat; TC/HDL-c: total cholesterol-to-HDL cholesterol ratio; TG/HDL-c: triglyceride-to-HDL cholesterol ratio; WtHR: waist-to-height ratio.

**Table 2:** Anthropometric, vascular, metabolic, and lipid profile according to residual atherogenic dyslipidemia status.

Variable	TG/HDL-c < 3.0 (n = 152,727)	TG/HDL-c ≥ 3.0 (n = 13,281)	*p*-Value
Age (years)	36.61 ± 9.72	41.29 ± 10.01	<0.001
Male sex, n (%)	70,973 (46.5)	11,023 (83.0)	<0.001
BMI (kg/m^2^)	22.38 ± 1.66	23.21 ± 1.51	<0.001
Waist circumference (cm)	78.12 ± 8.61	87.13 ± 8.42	<0.001
Waist-to-height ratio	0.466 ± 0.049	0.515 ± 0.047	<0.001
METS-VF	5.20 ± 0.63	6.68 ± 0.58	<0.001
Systolic blood pressure (mmHg)	117.91 ± 13.32	128.04 ± 13.56	<0.001
Diastolic blood pressure (mmHg)	74.02 ± 8.95	82.37 ± 9.18	<0.001
Fasting glucose (mg/dL)	90.64 ± 10.84	100.53 ± 14.46	<0.001
Total cholesterol (mg/dL)	184.08 ± 33.88	228.32 ± 41.72	<0.001
LDL cholesterol (mg/dL)	112.09 ± 30.81	139.82 ± 34.65	<0.001
HDL cholesterol (mg/dL)	54.55 ± 6.92	47.26 ± 7.56	<0.001
Triglycerides (mg/dL)	77.45 ± 28.53	211.49 ± 87.54	<0.001
Non-HDL cholesterol (mg/dL)	130.40 ± 36.99	170.11 ± 43.74	<0.001
TC/HDL-c ratio	3.38 ± 0.76	5.02 ± 1.12	<0.001
LDL-c/HDL-c ratio	2.06 ± 0.63	3.10 ± 0.89	<0.001
TyG index	8.31 ± 0.42	9.41 ± 0.53	<0.001
Hypertriglyceridemia, n (%)	4803 (3.1)	8475 (63.8)	<0.001
Low HDL cholesterol, n (%)	11,484 (7.5)	7441 (56.0)	<0.001
Impaired fasting glucose, n (%)	9553 (6.3)	2565 (19.3)	<0.001
Elevated blood pressure, n (%)	24,383 (16.0)	5332 (40.1)	<0.001
Central adiposity (WtHR ≥ 0.50), n (%)	14,276 (9.3)	5313 (40.0)	<0.001

Data are presented as mean ± standard deviation or absolute frequency and percentage. BMI: body mass index; HDL-c: high-density lipoprotein cholesterol; LDL-c: low-density lipoprotein cholesterol; METS-VF: Metabolic Score for Visceral Fat; TC/HDL-c: total cholesterol-to-HDL cholesterol ratio; TG/HDL-c: triglyceride-to-HDL cholesterol ratio; TyG: triglyceride-glucose index; WtHR: waist-to-height ratio.

**Table 3:** Lipid and cardiometabolic profile according to METS-VF quartiles.

Variable	Q1 METS-VF	Q2 METS-VF	Q3 METS-VF	Q4 METS-VF	*p*-Value
Age (years)	30.84 ± 6.82	35.27 ± 7.91	39.12 ± 8.64	42.73 ± 9.22	<0.001
Male sex, n (%)	14,528 (35.0)	18,094 (43.6)	22,661 (54.6)	26,713 (64.4)	<0.001
BMI (kg/m^2^)	20.89 ± 1.12	21.94 ± 1.08	22.98 ± 1.04	24.01 ± 0.79	<0.001
Waist circumference (cm)	71.24 ± 5.81	76.03 ± 5.62	81.28 ± 5.71	86.82 ± 5.88	<0.001
Waist-to-height ratio	0.427 ± 0.031	0.456 ± 0.028	0.487 ± 0.029	0.521 ± 0.031	<0.001
Systolic blood pressure (mmHg)	112.42 ± 11.54	116.71 ± 12.03	121.03 ± 12.78	124.71 ± 13.45	<0.001
Diastolic blood pressure (mmHg)	70.11 ± 7.54	73.22 ± 8.03	76.15 ± 8.48	79.28 ± 8.91	<0.001
Fasting glucose (mg/dL)	86.88 ± 8.21	89.73 ± 9.01	93.21 ± 10.64	96.02 ± 12.33	<0.001
Total cholesterol (mg/dL)	176.52 ± 31.65	183.71 ± 33.92	191.58 ± 36.08	198.66 ± 38.22	<0.001
LDL cholesterol (mg/dL)	106.34 ± 28.55	111.47 ± 29.94	117.93 ± 31.52	121.52 ± 33.74	<0.001
HDL cholesterol (mg/dL)	57.21 ± 7.32	54.83 ± 6.91	52.61 ± 6.84	50.98 ± 7.11	<0.001
Triglycerides (mg/dL)	67.14 ± 24.53	79.65 ± 29.88	95.82 ± 38.66	111.64 ± 56.77	<0.001
Non-HDL cholesterol (mg/dL)	119.31 ± 32.45	128.88 ± 34.73	138.97 ± 37.21	147.68 ± 39.85	<0.001
TG/HDL-c ratio	1.23 ± 0.54	1.58 ± 0.73	1.96 ± 1.02	2.41 ± 1.51	<0.001
TyG index	8.04 ± 0.35	8.28 ± 0.41	8.53 ± 0.47	8.81 ± 0.58	<0.001
Elevated TG/HDL-c ratio ≥ 3.0, n (%)	1121 (2.7)	2487 (6.0)	3121 (7.5)	6552 (15.8)	<0.001
Hypertriglyceridemia, n (%)	1204 (2.9)	2860 (6.9)	3692 (8.9)	6387 (15.4)	<0.001
Impaired fasting glucose, n (%)	1412 (3.4)	2785 (6.7)	3845 (9.3)	5106 (12.3)	<0.001
Elevated blood pressure, n (%)	4153 (10.0)	6472 (15.6)	8721 (21.0)	10,369 (25.0)	<0.001
Central adiposity (WtHR ≥ 0.50), n (%)	1038 (2.5)	3482 (8.4)	5902 (14.2)	9167 (22.1)	<0.001

Data are presented as mean ± standard deviation or absolute frequency and percentage. HDL-c: high-density lipoprotein cholesterol; LDL-c: low-density lipoprotein cholesterol; METS-VF: Metabolic Score for Visceral Fat; TG/HDL-c: triglyceride-to-HDL cholesterol ratio; TyG: triglyceride-glucose index; WtHR: waist-to-height ratio.

**Table 4:** Residual atherogenic lipid abnormalities according to central adiposity status.

Variable	WtHR < 0.50 (n = 146,419)	WtHR ≥ 0.50 (n = 19,589)	*p*-Value
Age (years)	35.92 ± 9.31	44.95 ± 8.84	<0.001
Male sex, n (%)	66,835 (45.6)	15,161 (77.4)	<0.001
BMI (kg/m^2^)	22.16 ± 1.55	24.04 ± 1.02	<0.001
Waist circumference (cm)	76.31 ± 6.72	90.25 ± 6.11	<0.001
METS-VF	5.01 ± 0.54	6.83 ± 0.59	<0.001
Systolic blood pressure (mmHg)	117.01 ± 13.02	131.34 ± 12.95	<0.001
Diastolic blood pressure (mmHg)	73.88 ± 8.72	80.75 ± 8.91	<0.001
Fasting glucose (mg/dL)	90.48 ± 10.94	98.54 ± 13.28	<0.001
Total cholesterol (mg/dL)	185.84 ± 34.72	201.03 ± 39.21	<0.001
LDL cholesterol (mg/dL)	113.21 ± 30.95	122.54 ± 34.02	<0.001
HDL cholesterol (mg/dL)	54.13 ± 7.15	52.75 ± 7.80	<0.001
Triglycerides (mg/dL)	86.72 ± 50.45	98.55 ± 58.89	<0.001
Non-HDL cholesterol (mg/dL)	131.71 ± 36.45	148.28 ± 40.11	<0.001
TG/HDL-c ratio	1.69 ± 1.14	2.18 ± 1.64	<0.001
TyG index	8.34 ± 0.43	8.71 ± 0.58	<0.001
Elevated TG/HDL-c ratio ≥ 3.0, n (%)	10,688 (7.3)	2593 (12.8)	<0.001
Hypertriglyceridemia, n (%)	10,540 (7.2)	2738 (14.0)	<0.001
Low HDL cholesterol, n (%)	15,614 (10.7)	3311 (16.9)	<0.001
Impaired fasting glucose, n (%)	9459 (6.5)	2659 (13.6)	<0.001
Elevated blood pressure, n (%)	23,028 (15.7)	6687 (34.1)	<0.001

Data are presented as mean ± standard deviation or absolute frequency and percentage. HDL-c: high-density lipoprotein cholesterol; LDL-c: low-density lipoprotein cholesterol; METS-VF: Metabolic Score for Visceral Fat; TG/HDL-c: triglyceride-to-HDL cholesterol ratio; TyG: triglyceride-glucose index; WtHR: waist-to-height ratio.

**Table 5:** Sex-specific differences in lipid, adiposity, and cardiometabolic risk markers.

Variable	Men (n = 81,996)	Women (n = 84,012)	*p*-Value
Age (years)	37.84 ± 9.92	36.17 ± 9.68	<0.001
BMI (kg/m^2^)	22.78 ± 1.63	22.13 ± 1.65	<0.001
Waist circumference (cm)	82.94 ± 8.11	74.84 ± 7.12	<0.001
Waist-to-height ratio	0.482 ± 0.046	0.458 ± 0.048	<0.001
METS-VF	5.63 ± 0.73	5.01 ± 0.61	<0.001
Systolic blood pressure (mmHg)	122.81 ± 13.34	114.72 ± 12.34	<0.001
Diastolic blood pressure (mmHg)	77.18 ± 8.92	72.25 ± 8.61	<0.001
Fasting glucose (mg/dL)	93.44 ± 11.83	89.46 ± 11.21	<0.001
Total cholesterol (mg/dL)	189.95 ± 36.52	185.34 ± 35.11	<0.001
LDL cholesterol (mg/dL)	115.88 ± 32.14	112.78 ± 31.12	<0.001
HDL cholesterol (mg/dL)	50.71 ± 6.42	57.19 ± 6.53	<0.001
Triglycerides (mg/dL)	101.83 ± 58.92	75.60 ± 39.48	<0.001
Non-HDL cholesterol (mg/dL)	139.24 ± 38.52	128.15 ± 35.32	<0.001
TG/HDL-c ratio	2.19 ± 1.42	1.40 ± 0.91	<0.001
TC/HDL-c ratio	3.89 ± 0.96	3.24 ± 0.78	<0.001
TyG index	8.55 ± 0.52	8.24 ± 0.41	<0.001
Elevated TG/HDL-c ratio ≥ 3.0, n (%)	9983 (12.2)	3298 (3.9)	<0.001
Hypertriglyceridemia, n (%)	10,250 (12.5)	3028 (3.7)	<0.001
Low HDL cholesterol, n (%)	2460 (3.0)	16,465 (19.7)	<0.001
Impaired fasting glucose, n (%)	7144 (8.7)	4974 (5.9)	<0.001
Elevated blood pressure, n (%)	18,973 (23.1)	10,742 (12.8)	<0.001
Central adiposity (WtHR ≥ 0.50), n (%)	12,531 (15.3)	7058 (8.4)	<0.001
Current smokers, n (%)	29,614 (36.1)	27,160 (32.3)	<0.001
Physical inactivity, n (%)	26,961 (32.9)	31,973 (38.1)	<0.001

Data are presented as mean ± standard deviation or absolute frequency and percentage. HDL-c: high-density lipoprotein cholesterol; LDL-c: low-density lipoprotein cholesterol; METS-VF: Metabolic Score for Visceral Fat; TC/HDL-c: total cholesterol-to-high-density lipoprotein cholesterol ratio; TG/HDL-c: triglyceride-to-high-density lipoprotein cholesterol ratio; TyG: triglyceride-glucose index; WtHR: waist-to-height ratio.

**Table 6:** Prevalence of residual atherogenic dyslipidemia according to lifestyle-related factors.

Variable	Elevated TG/HDL-c Ratio ≥ 3.0 n (%)	Hypertriglyceridemia n (%)	Low HDL Cholesterol n (%)	*p*-Value
Physical activity				<0.001
Physically active	412 (0.4)	1865 (1.7)	7248 (6.5)	
Physically inactive	12,869 (35.3)	11,413 (31.0)	11,677 (31.5)	
Mediterranean diet adherence				<0.001
High adherence	842 (0.8)	2551 (2.5)	8442 (8.1)	
Low adherence	12,439 (26.7)	10,727 (22.9)	10,483 (22.4)	
Smoking status				<0.001
Non-smokers	5598 (6.0)	6129 (6.6)	9416 (10.1)	
Current smokers	7683 (11.0)	7149 (10.2)	9509 (13.6)	
Alcohol consumption				<0.001
No habitual consumption	4711 (5.9)	5003 (6.2)	8281 (10.3)	
Regular consumption	8570 (9.9)	8275 (9.5)	10,644 (12.2)	

Data are presented as absolute frequency and percentage. HDL-c: high-density lipoprotein cholesterol; TG/HDL-c: triglyceride-to-high-density lipoprotein cholesterol ratio.

**Table 7:** Multivariable logistic regression models for elevated TG/HDL-c according to different adiposity measures.

Variable	Model 1: BMI OR (95% CI)	Model 2: WtHR ≥ 0.50 OR (95% CI)	Model 3: METS-VF OR (95% CI)
Male sex	4.62 (4.40–4.86)	5.01 (4.76–5.26)	4.13 (3.89–4.38)
Age, per year	0.994 (0.991–0.996)	0.996 (0.994–0.999)	0.991 (0.988–0.993)
BMI, per kg/m^2^	1.13 (1.11–1.15)	—	—
WtHR ≥ 0.50	—	0.96 (0.91–1.03)	—
METS-VF, per unit	—	—	1.30 (1.24–1.37)
Impaired fasting glucose	1.25 (1.16–1.34)	1.26 (1.18–1.35)	1.26 (1.17–1.35)
Elevated blood pressure	1.26 (1.19–1.33)	1.29 (1.22–1.36)	1.27 (1.21–1.35)
Physical inactivity	104.37 (93.08–117.04)	105.04 (93.70–117.76)	104.16 (92.89–116.80)
Low Mediterranean diet adherence	1.99 (1.82–2.18)	2.03 (1.85–2.22)	2.00 (1.83–2.19)
Current smoking	1.80 (1.72–1.89)	1.73 (1.65–1.81)	1.74 (1.66–1.82)

Model 1 included BMI; Model 2 included waist-to-height ratio (WtHR); and Model 3 included METS-VF. Adiposity-related measures were entered in separate models to minimize collinearity. All models were adjusted for the same demographic, metabolic, and lifestyle-related covariates. OR: odds ratio; CI: confidence interval; BMI: body mass index; METS-VF: Metabolic Score for Visceral Fat.

## Data Availability

The information generated and examined during this study is securely maintained within the institutional repositories of ADEMA University School. Requests for access to anonymised datasets may be evaluated by the corresponding author when appropriately justified and when consistent with ethical requirements and applicable legislation related to data privacy and protection.
